# Effects of tadalafil to prevent injury on corpus cavernosum after vascular or nervous peri-prostatic bundle injury. Experimental model in rats^1^

**DOI:** 10.1590/s0102-865020190090000001

**Published:** 2019-11-28

**Authors:** Antônio Carlos de Toledo, Paulo Roberto Kawano, Hamilto Akihissa Yamamoto, Rodrigo Guerra, Fernando Ferreira Gomes, Pedro Ivo Rochetti Pajolli, João Luiz Amaro, Luiz Eduardo Macedo Cardoso, Francisco José Sampaio

**Affiliations:** I PhD, Postgraduate Program in Bases General Surgery, Botucatu School of Medicine, Universidade Estadual Paulista (UNESP), Botucatu-SP, Brazil. Acquisition, analysis and interpretation of data; technical procedures; histopathological examinations; manuscript preparation; II Associated Professor, Department of Urology, Botucatu School of Medicine, UNESP, Botucatu-SP, Brazil. Design, intellectual and scientific content of the study; manuscript writing, critical revision, final approval; III Assistant Professor, Department of Urology, Botucatu School of Medicine, UNESP, Botucatu-SP, Brazil. Statistics analysis, manuscript writing, critical revision; IV PhD, Department of Urology, Botucatu School of Medicine, UNESP, Botucatu-SP, Brazil. Acquisition, analysis and interpretation of data; manuscript writing; V Fellow Master degree, Postgraduate Program in Bases General Surgery, Botucatu School of Medicine, UNESP, Botucatu-SP, Brazil. Acquisition, analysis and interpretation of data; technical procedures; VI Assistant Physician, Department of Urology, Botucatu School of Medicine, UNESP, Botucatu-SP, Brazil. Acquisition of data, technical procedures; VII Full Professor, Department of Urology, Botucatu School of Medicine, UNESP, Botucatu-SP, Brazil. Conception and design of the study, critical revision, final approval; VIII Associate Professor; Urogenital Research Unit, Universidade Estadual do Rio de Janeiro (UERJ), Brazil. Conception and design of the study, histopathological examinations, critical revision, final approval; IX Full Professor, Urogenital Research Unit, UERJ, Rio de Janeiro-RJ, Brazil. Critical revision, final approval

**Keywords:** Collagen, Erectile Dysfunction, Elastin, Tadalafil, Rats

## Abstract

**Purpose:**

To evaluate the effects of tadalafil (TD) in preventing histological alterations of the corpus cavernosum caused by isolated lesions of cavernous nerve (ILCN) and artery (ILCA) in rats.

**Methods:**

Fifty male Wistar rats were randomly assigned in five groups: G1: control; G2: bilateral ILCN; G3: bilateral ILCA; G4: ILCN+TD; G5: ILCA+TD. The cavernous bodies were submitted to histomorphometry, immunohistochemistry and biochemical analysis.

**Results:**

Nerve density was significantly higher in G2 and G4 compared to control (22.62±2.84 and 19.53±3.47 vs. 15.72±1.82; respectively, p<0.05). Smooth muscle density was significantly lower in G2 and G3 in comparison to G1 (12.87±1.90 and 18.93±1.51 vs. 21.78±1.81, respectively; p<0.05). A significant decrease in the sinusoidal lumen area was observed in G2 compared to controls (5.01±1.62 vs. 9.88±3.66, respectively; p<0.05) and the blood vessel density was increased in G2 and G3 (29.32±4.13 e 20.80±2.47 vs. 10.13±2.71, p<0.05). Collagen density was higher in G3 compared to G1 (93.76±15.81 vs. 64.59±19.25; p<0.05).

**Conclusions:**

Histomorphometric alterations caused by ILCN were more intense than those produced by vascular injury, but the collagen analyses showed more fibrosis in animals with ILCA. TD was effective in preventing the majority of the alterations induced by the periprostatic bundle injury.

## Introduction

Prostate Cancer (PC) is the most prevalent non-cutaneous solid tumor in men over 50 years, being responsible for about 40% of tumors affecting males, and represents the second cause of cancer-specific mortality in the United States^1^.

Currently, radical prostatectomy (RP) with preservation of neurovascular bundles is the main surgical treatment for the localized disease^2^. However, its most common complication is erectile dysfunction (ED), which may happen in 30 to 50% of cases^3^ ^–^ ^5^, imposing a negative impact on the quality of life of these patients^6^. The main cause of ED after RP is neuropraxia of the cavernous nerve, due to inflammatory process resulting from the prostate dissection, or thermal lesion caused by electrocautery^7^. This nervous injury may lead to microstructural derangement of the erectile tissue, characterized by increase in the production of collagen, and vascular remodeling, with consequent impairment of the smooth musculature^8^ ^–^ ^10^. The vascular injury may result in decreased oxygenation of the erectile tissue, contributing to worsen ED^11^ ^,^ ^12^.

Some authors^13^ ^,^ ^14^ have suggested that using phosphodiesterase-5 inhibitors (IPDE-5) could reduce the occurrence of ED after surgery for PC; however, the specific mechanism of their action is not well established in this setting. In theory, IPDE-5 could improve oxygenation of the corpus cavernosum and hence prevent overt tissue alterations related with nervous and vascular damage during the prostatectomy^13^ ^,^ ^14^.

Although further studies are required to evaluate the real impact of nervous and/or vascular lesions after RP, they are difficult to be carried out in humans. On the other hand, rat models are well accepted in studying structural alterations that cause ED after RP, once pelvic innervation and anatomy of cavernous nerves have been widely studied in these animals^15^ ^,^ ^16^.

So, in order to better understand these questions, the present study aims to evaluate the effects of tadalafil in preventing histological alterations of the corpus cavernosum caused by isolated lesions of cavernous nerve and artery, using a rat experimental model.

## Methods

A total of 50 male Wistar rats, weighing between 250 and 350 grams, were randomly divided in five groups: Group 1 (G1: surgical control, n=10): the animals underwent surgical intervention for dissection and identification of the peri-prostatic neurovascular bundle. Group 2 (G2: Isolated nervous lesion, n=10): after identification and dissection of the neurovascular bundle, a 5mm segment of the cavernous nerve was dried out bilaterally, as described by Quinlan and User^16^. Group 3 (G3: Isolated vascular lesion, n=10): surgical ligature and section of the cavernous artery, bilaterally. Group 4 (G4: Isolated nervous lesion + Tadalafil, n=10): animals had the same procedure of G2 and also received tadalafil for a period of 45 days. And Group 5 (G5: Isolated vascular lesion + Tadalafil, n=10): animals were submitted to the same procedure of G3 and also received tadalafil for 45 days. The study was previously approved by the Ethics Committee on Animal Experiments, from Universidade Estadual Paulista, Botucatu School of Medicine (protocol number: 756/2009).

### Intervention

For the surgical procedure, each animal was anesthetized with ketamine (0.30 mg/kg) and xylazine (0.25-0.30 mg/kg), administered intramuscularly. After abdominal incision, dissection of the structures of interest was performed with microsurgical instruments under x6 magnification. The proceedings were done in accordance to each group of study and, after surgery, the animals were maintained in individual cages. In groups G4 and G5, from the third post-operative day, tadalafil was administered by gavage, at the dosage of 5mg/kg/day, diluted in 10% glucose, for a period of 45 days. This dosage of tadalafil corresponds to 20 mg/day in humans^17^.

The medication was interrupted three days before the euthanasia, to allow the clearance of the drug (“wash out”), and the penis was removed *en bloc*. The proximal segment, which extends from the bone to the peri-prostatic bundle, was fixated in 10% buffered formalin during 48 hours and subsequently sent to histological processing for inclusion in paraffin.

### Histomorphometric analysis

The cross-sectional area of corpus cavernosum was standardized among the different study groups, leading to homogeneity in histological process. Sections of 5μm were stained with hematoxylin-eosin (HE) for analysis of their section area, volume of muscular fibers and sinusoidal volume. Collagen was analyzed by *Picrosirius red* staining, and elastic fibers were characterized by immunofluorescence.

Histological images were digitalized with a magnification of x200, using a video camera coupled to a polarized optic microscope (LEICA DMLB^®^). The measurement of smooth muscle fibers and sinusoidal spaces was done in five random fields using a grid of 100 points created with Image J^®^ software, and the final result was expressed as a percentage. All the transversal area of the corpus cavernosum, excluding the tunica albuginea, was utilized as the reference space. Tubulin beta-3 marker for axons was used for the evaluation of nerve density in the fibrous penile septum.

The density of blood vessels in the cavernous trabeculae was analyzed by immunohistochemistry using anti-CD-31 antibody, and results were expressed in number of vessels/mm^2^. A cellular proliferation marker - Monoclonal Mouse Anti-Proliferating Cell Nuclear Antigen (PCNA), was used to evaluate cellular proliferation in the cavernous body. The results were expressed in positive nuclei/mm^2^. The subendothelial smooth muscle layer was included in this evaluation.

For quantification of collagen fibers, the surface area occupied by collagen fibers was measured and expressed in μm^2^.

Density of elastic fibers was obtained qualitatively through immunofluorescence^18^ enabling for evaluation of their distribution and organization in the cavernous body.

### Statistical analysis

For collagen evaluation, non-parametric variance analysis was applied, complemented with Dunn's multiple comparison test^20^. For the others variables, a technique of parametric variance analysis was used, with Bonferroni's multiple comparisons test. All multiple comparisons were analyzed considering a 5% significance level.

## Results

There were no adverse effects of tadalafil such as lethargy or priapism, and all animals presented adequate weight gain in all groups during the study.

Histological analyses showed that nerve density in penile trabecular tissue was significantly higher in G2 and G4 compared to controls. There was no statistical difference among the other groups.

Smooth muscle density in corpus cavernosum was significantly lower in G2 and G3 in comparison to G1. However, animals with neuro-vascular lesions treated with tadalafil (G4 and G5) presented smooth muscle distribution similar to the control group.

In the nervous lesion group (G2), we observed a significant decrease in sinusoidal lumen area compared to the control group. There was no statistical difference among other groups, specifically in the animals with nervous lesion treated with tadalafil (G3).

Density of blood vessels in the cavernous trabecula increased significantly in animals with nervous and vascular lesions (G2 and G3) compared to control group (Table 1). However, this effect was not observed in animals treated with tadalafil (G4 and G5).

**Table 1:** Description of the histomorphometric variables in the different studied groups.

Variables	Groups					Statistical analysis
	G1 (n = 10)	G2 (n = 10)	G3 (n = 10)	G4 (n = 10)	G5 (n = 10)	
Cross-sectional area of the penis(1)	8.17 (0.59)	8.81 (1.24)	8.75 (0.71)	8.75 (1.04)	8.26 (0.78)	p>0.05
Nerve density in the penile trabecula(1)	15.72 (1.82)¥	22.62 (2.84)	16.72 (2.08)	19.53 (3.47)	15.67 (2.61)	p<0.001
Volume of Smooth Muscle in CC(1)	21.78 (1.81)#	12.87 (1.90)	18.93 (1.51)	19.49 (2.49)	19.37 (1.89)	p<0.05
Volume of sinusoidal space in CC(1)	9.80 (3.66)&	5.01 (1.62)	6.33 (1.84)	8.01 (3.29)	8.09 (2.41)	p<0.001
Vessel density in penile trabecula(1)	10.13 (2.71)θ	29.32 (4.13)	20.80 (2.47)	12.89 (4.63)	9.89 (2.51)	p<0.001
Cell Proliferation (PCNA)(2)	45.82 (11.21; 70.92)	517.73β (107.80; 788.65)	121.99 (49.79; 262.41)	269.65 (175.89; 560.28)	35.46 (12.77; 130.50)	p<0.001
Collagen level (1)	64.59 (19.24)	47.48 (15.31)	93.76 (15.81)$	76.26 (11.18)	63.11 (21.11)	p<0.001

CC: Corpus Cavernosum. PCNA: Anti-Proliferating Cell Nuclear Antigen.

(1) Mean (standard deviation).

(2) Median (minimum value; maximum value).

¥ (p< 0.001) G1 X (G2, G4).

# (p< 0.05) G1 X (G2, G3).

& (p< 0.001) G1 X G2.

θ (p< 0.001) G1 X (G2, G3).

β (p< 0.001) G1 X G2 X G3 X G4 X G5.

$ (p< 0.001) G3 X (G1, G2).

There was a significant increase in cellular proliferation in G2, G3 and G4 compared to control. However, the use of tadalafil resulted in an important decrease in cellular proliferation in G5.

Collagen fiber quantification in corpus cavernosum was significantly higher in G3 (vascular lesion) compared to G1 and G2. There was no statistical difference among animals treated with tadalafil (G4 and G5) in relation to control.

There was a significant reduction in number, as well as structural disorganization, of elastic fibers of corpus cavernosum in G2 and G3 (nervous or vascular lesion). However, in animals treated with tadalafil (G4 and G5), this parameter had similar behavior of controls.

All data presented above are summarized in Table 1.

## Discussion

Currently, radical prostatectomy (RP) represents an effective and curative intervention for clinically localized prostate cancer, achieving excellent long-term cancer control rates^2^. However, erectile dysfunction remains a major complication of RP, being responsible, regardless of the current refinements in surgical techniques, for significant impairment to the patient's sexual life, impacting on overall quality of life after treatment^3^ ^,^ ^4^.

According to the literature, the neurogenic component probably plays a fundamental role in the pathogenesis of sexual dysfunction (SD) after RP^7^. Developments in the surgical technique, such as the preservation of neurovascular bundles, were proposed to minimize this complication^7^. In the present study, we observed a significant increase of nerve density in the penile trabecular tissue in G2 (nerve lesion) and G4 (nerve lesion plus tadalafil) when compared to the control group. This fact probably occurred due a compensatory tissue reaction as a response to the lesion in the nervous plexus in these groups. This finding suggests that tadalafil was unable to protect these animals from the effects of nervous injury.

Different authors have demonstrated a decrease in smooth muscle density and sinusoidal volume in the corpus cavernosum (CC) of patients with sexual dysfunction after RP, probably due to an ischemic process triggered by the peri-prostatic bundle lesion^19^ ^–^ ^21^. In our study, we observed a significant reduction in smooth muscle density in the CC of animals submitted to both nervous and vascular isolated lesions (G2 and G3), especially in G2. However, the protective action of tadalafil was evident in treated groups (G4 e G5), which had similar smooth muscle density to the control group.

As expected, we noted a significant decrease in CC sinusoidal space volumes in the animals submitted to lesion of the periprostatic nervous bundle (G2). However, there was no statistical difference between controls and animals with nerve injury treated with tadalafil (G4), demonstrating that this drug did show a protective effect in the tissue remodeling process caused by the nervous damage.

Vascular density in the penile trabecular space was significantly higher in G2 and G3, probably due to a compensatory process triggered by the CC ischemia secondary to the peri-prostatic neurovascular bundle lesion. Despite this, in treated groups (G4 and G5), we observed a protective effect of tadalafil, which reduced the vascular proliferation rates closely to control. Many studies have suggested that the vasodilatation induced by drugs like phosphodiesterase E-5 inhibitors (IPDE-5) could protect the intra-cavernous erectile tissue from damage derived from the lesion of the periprostatic bundles after radical prostatectomy^13^ ^,^ ^14^ ^,^ ^17^.

In our series, we observed a considerable impact of periprostatic bundle injuries on the cellular proliferation, demonstrating that there was an important structural remodeling process in the CC of these animals. This effect was more remarkable in G2 (nervous lesion). It is important to note that animals with nervous injury treated with tadalafil (G4), in absolute numbers, showed a 47% reduction in the cellular proliferation of CC, when compared to G2. Similar behavior was observed among the animals with isolated vascular lesion, in which the drug treatment resulted in a significant decrease in cellular proliferation rates (13%). These results suggest a protective effect of tadalafil in reducing the cellular proliferation rates in CC of animals with periprostatic neurovascular bundle injuries.

Some authors have reported that collagen is responsible for up to 63% of the composition of cavernous trabecular tissue in normal rats^22^. Additionally, controlled studies showed an increase of collagen and a decrease of smooth muscle composition in CC of men with erectile dysfunction^11^ ^,^ ^23^. In our results, we observed a significant increase of collagen in G3, but not in G2, suggesting that vascular injury could be worse than nervous. However, animals with isolated vascular lesion treated with tadalafil (G5) presented similar results to controls, showing that this drug could have a protective effect on CC. This is in line with previous human studies that have suggested that peri-prostatic bundle vascular injury induces development of fibrosis in the CC of post-prostatectomy patients^11^ ^,^ ^23^. Unfortunately, this protective effect could not be demonstrated in animals with isolated nerve damage.

It is known that the composition of elastic fibers of CC has an important hole in the erection process^11^ ^,^ ^19^. In our series, it was evident a major structural disorganization of these fibers in the groups with peri-prostatic bundle lesions (G2 and G3). However, in opposition to the other evaluated histological parameters, the results of animals treated with tadalafil were similar to the controls, suggesting that this drug was able to prevent the remodeling tissue process postoperatively in the CC.

## Conclusions

We demonstrated that, despite the histomorphometric alterations of the isolated nervous lesion being worse than those produced by vascular injury, the collagen density analyses showed more fibrosis in the corpus cavernosum of animals with vascular lesion. In this model, tadalafil was effective in preventing the majority of the histomorphometric alterations induced by the periprostatic bundle injury, and also in minimizing the remodeling tissue process in the erectile tissue.
